# Association between Working Hours and Self-Rated Health

**DOI:** 10.3390/ijerph17082736

**Published:** 2020-04-15

**Authors:** Jongha Jeon, Wanhyung Lee, Won-Jun Choi, Seunghon Ham, Seong-Kyu Kang

**Affiliations:** 1 College of Medicine, Gachon University, Incheon 21565, Korea; 98whdgk@naver.com; 2 Department of Occupational and Environmental Medicine, Gil Medical Center, Gachon University College of Medicine, Incheon 21565, Korea; wanhyung@gmail.com (W.L.); wjchoi@gachon.ac.kr (W.-J.C.); shham@gachon.ac.kr (S.H.)

**Keywords:** working hours, self-rated health, KNHANES

## Abstract

This study compared the association between working hours and self-rated health (SRH) according to sex, socioeconomic status, and working conditions. In all, 25,144 participants were selected from the Korea National Health and Nutrition Examination Survey (KNHANES), conducted from 2010 to 2018. The risks of poor SRH, according to working hours, were investigated using multiple logistic regression. Both short and long working hours were associated with poor SRH. Men working short hours and women working long hours were at risk of poor SRH. Workers with fewer than nine years of education were at risk of poor SRH when working short hours, whereas workers with more than nine years of education were at risk when working long hours. Similarly, simple laborers were at risk of poor SRH when working short hours, while managers and professional workers were at risk when working long hours. When working for short hours, paid employees were at risk of poor SRH. Workers with a non-fixed work schedule showed no risk of poor SRH when working long or short hours. In conclusion, workers working short hours with low education and workers working long hours with high education were at risk of poor SRH. Working conditions were significantly related to the association between SRH and working hours.

## 1. Introduction

The 2018 report by the Organization for Economic Cooperation and Development (OECD) indicated that the Republic of Korea (hereinafter Korea) had the third-longest number of working hours (1933 h per year) among countries in the OECD, following Mexico (2148 h) and Costa Rica (2121 h) [1]. Long working hours are a risk factor for workers’ health, leading to depressive symptoms [2,3,4], unhealthy weight gain [2,5], poor sleep condition [3,6], poor cognitive function [7], and anxiety [3]. Moreover, the associations between long working hours and several chronic diseases such as coronary heart disease [3,8], stroke [9], metabolic syndrome [10], and injury [11] were also reported.

Self-rated health (SRH) was verified as a predictor of mortality [12,13] and working sustainability [14]; owing to its simplicity and integrity, it can be employed as a general marker for many health-related factors [15]. Several studies investigated the associations between working hours and SRH. Two of these studies revealed that long working hours [16,17] and short working hours [17] were associated with poor SRH, more commonly among women. Such an association was also reported in young workers [18]. However, most of the studies investigated the trends of SRH with the stratification of only sex [16,18,19]. 

However, several studies showed that socioeconomic status (SES) may be related to the association between working conditions and health. According to a study, the increase in type 2 diabetes based on long working hours was greater in individuals with low SES [20]. In another study, only non-manual workers had a risk of obesity based on shift and night work [5].

Thus, to determine how personal, social, and work traits influence SRH, we investigated the trends in SRH according to working hours with stratification of sex, SES, and working conditions, employing the survey data representative of workers in Korea.

## 2. Materials and Methods 

### 2.1. Data Collection and Study Participants

**Figure 1:**
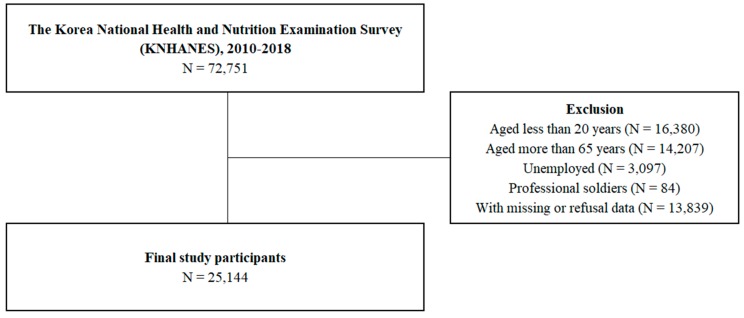
Schematic diagram depicting study population.

This cross-sectional study was based on the Korea National Health and Nutrition Examination Survey (KNHANES) conducted from 2010 to 2018, which included health behavioral status, SES, and working conditions as variables. The Korea Centers for Disease Control and Prevention conducted these surveys. In KNHANES, the sample design and size are estimated to represent the entire Korean population, so that the results can be employed to represent the overall population in Korea in each survey year [21]. The rates of participation in 2010–2018 ranged from 76.6% to 80.8%. Figure 1 shows the exclusion criteria used in ultimately selecting 25,144 study participants.

### 2.2. Measurement of Weekly Working Hours 

Participants were asked about their average working hours per week, including night work and extra working time; weekly working hours were grouped into four categories: <20 h, 20–39 h, 40–59 h, and ≥60 h. 

### 2.3. Self-Rated Health (SRH) 

Participants were asked to evaluate their health using a five-point scale; “very good,” “good,” and “fair” were defined as good SRH, whereas “very poor” and “poor” were defined as poor SRH.

### 2.4. Categorizations of Socioeconomic Status (SES) and Working Conditions

SES (household income, education, and occupation) and working conditions (type of work and work schedule) were categorized for stratification. Household income was classified into four quartiles based on national statistics. Education was classified into four levels according to the Korean school system: “fewer than six years (elementary school),” “fewer than nine years (junior high school),” “fewer than 12 years (high school),” and “more than 12 years (college).” The occupation was classified into six categories: simple labor (Major Group 9: elementary occupations), craft, plant and machine operators, and assemblers (Major Group 7: craft and related trade workers and Major Group 8: plant and machine operators and assemblers), skilled agricultural, forestry, and fishery (Major Group 6: skilled agricultural, forestry, and fishery workers), service and sales (Major Group 5: service and sales workers), clerk (Group 4: clerical support workers), and manager and professional (Major Group 1: legislators, senior officials, and managers; Major Group 2: professionals; Major Group 3: technicians and associate professionals) based on the International Standard Classification of Occupations. Korea assigns more than 18 months of mandatory military duty to all male citizens (approximately 20–24 years old). Thus, Major Group 0: armed forces (professional soldier) was excluded due to heterogeneity in age and gender.

### 2.5. Measurements of Covariates

A previous study indicated that SRH was closely related to health behavioral factors (smoking and drinking), body mass index (BMI), and chronic diseases [22]. We selected covariates from both previous studies and backward stepwise selection. Smoking was classified into three categories: never, past, and current. Smokers who smoked fewer than 100 cigarettes throughout their lifetime were defined as never, and smokers who smoked before but quit were defined as past smokers. Drinking was classified into three categories: none, moderate, and severe. Drinkers who had no drinking history within the year were defined as none, and drinkers who drank five glasses (or more) of alcohol twice (or more) per week were defined as severe. Obesity was classified into three categories according to body mass index (BMI): underweight (<18.5), normal (<25), and obese (≥25). Three diseases were chosen as confounding factors to be adjusted: hypertension, diabetes, and depression.

### 2.6. Statistical Analysis

**Figure 2:**
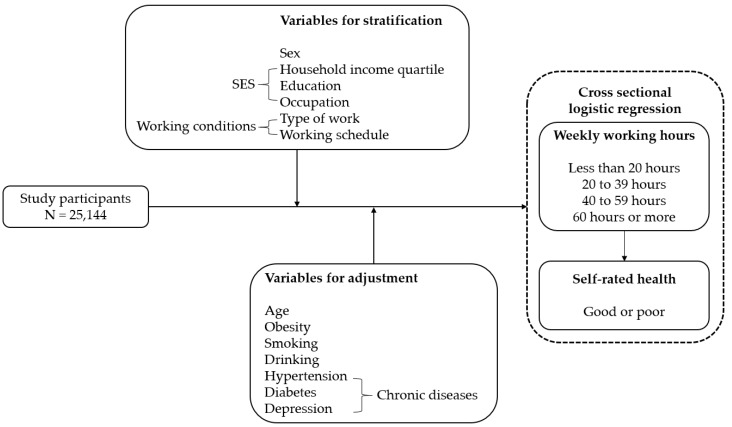
Analytical framework of current study.

SPSS software version 25 (IBM Corp., Armonk, NY, USA) was used for statistical analyses. A *p*-value <0.05 was considered statistically significant. The chi-square test was used to assess the relationships between participants’ working hours and other variables. Multiple logistic regression was used to estimate odds ratios (ORs) and 95% confidence intervals (CIs) to estimate the trends of poor SRH by working hours while adjusting for age, disease, smoking, drinking, and obesity. Sex, household income quartiles, education, occupational classification, type of work, and work schedule were stratified. Figure 2 shows the analytical framework of current study.

## 3. Results

After excluding the unemployed, individuals aged below 20 years and above 65 years, professional soldiers, and individuals who did not answer the questionnaire, a final sample of 25,144 participants was obtained (Figure 1). Table 1 presents the participants’ general characteristics. Men worked longer than women. Workers in the first quartile of household income worked shorter hours, and workers in the third and fourth quartiles of household income tended to work for 40–59 h per week, including most Korean workers. Similarly, workers with college-level education tended to work for 40–59 h per week. For sales and service workers, the ratio of workers working ≥60 h per week was the largest; however, for simple laborers, the ratio of workers working <20 h per week was the largest. Paid employees and fixed workers tended to work 40–59 h per week.

Table 2 shows the ORs (and 95% CIs) of poor SRH according to working hours with stratification of sex, SES, and working conditions. The ORs were calculated after adjusting for age, obesity, smoking, drinking, and diseases. The reference group was workers who worked 40–59 h per week since their SRH was better than other groups. With no stratification, there were risks of poor SRH in workers working <20 h (OR = 1.299, 1.140–1.481) and ≥60 h (OR = 1.289, 1.163–1.430) per week. Men working <20 h per week were at risk of poor SRH (OR = 1.384, 1.080–1.772) in contrast to women. There were significant risks of poor SRH in both men (OR = 1.224, 1.064–1.407) and women (OR = 1.401, 1.197–1.639) working ≥60 h per week. Workers in the fourth quartile of household income and working ≥60 h per week were at risk of poor SRH (OR = 1.462, 1.221–1.751). When working <20 h per week, there were significant risks of poor SRH in workers with elementary level (OR = 1.579, 1.143–2.181) and junior high-school level education (OR = 1.433, 1.024–2.007). However, when working ≥60 h per week, there were significant risks of poor SRH among workers with high school (OR = 1.273, 1.072–1.512) and college (OR = 1.321, 1.092–1.598) level education. Simple laborers working <20 h per week were at risk of poor SRH (OR = 1.596, 1.199–2.124). In contrast, there were significant risks of poor SRH among managers and professional workers (OR = 1.371, 1.048–1.793) and service and sales workers (OR = 1.219, 1.007–1.477) working ≥60 h per week. Paid employees working <20 h per week were at risk of poor SRH (OR = 1.436, 1.226–1.682) in contrast to employers or self-employed workers. There were significant risks of poor SRH in both paid employees (OR = 1.232, 1.064–1.427) and employers or self-employed workers (OR = 1.251, 1.048–1.493) working ≥60 h per week. There were significant risks of poor SRH in fixed workers working <20 h (OR = 1.405, 1.212–1.629) and ≥60 h (OR = 1.290, 1.149–1.449) per week, as opposed to non-fixed workers.

## 4. Discussion

After adjusting for several demographic and health-related factors, statistically significant ORs were estimated between long or short working hours and SRH. In summary, poor SRH was related to long and short working hours in the total population. Both men and women working long hours were at risk of poor SRH, were the magnitude was greater among women. Only men were at risk of poor SRH when working for short hours. Workers with low education were at risk of poor SRH when working short hours, whereas workers with high education were at risk of poor SRH when working long hours. Additionally, occupations associated with either high or low educational level showed an identical relation. Paid employees were at risk of poor SRH when working short hours as compared to employers or self-employed workers. Compared with non-fixed workers, workers with fixed work schedules were at risk of poor SRH when working short or long hours.

Several studies indicated the adverse effects of long working hours on SRH, which is attributed to high job demands accompanied by long working hours [16,17,18,19], and the results of the present study support them. Additionally, short working hours are related to precarious working conditions [23] (temporary or daily, part-time, or contingent jobs), which are associated with poor health [16,24,25,26,27].

The result that women working long hours showed poorer SRH than men working long hours can be explained by the “double burden” of household and working duties of women [16,28]. This is why service and sales workers, of which women’s proportion was 63.7% in the present study, showed an unhealthy association with long working hours. In contrast to women working short hours, the SRH of men working short hours was poor. This was inconsistent with previous Korean studies; however, they were methodologically different from the present study. A study, confirming that only women had poor SRH with short working hours, merged workers who worked <40 h per week as one category [16]. In another study, which showed no association between short working hours and SRH in both sexes, short working hours were assessed in only one category: 20–35 h per week [19]. These differences would lead to discordance. However, considering that workers working <20 h per week accounted for 8.5% of the total participants in the present study, such workers were not negligible; therefore, a category for these workers was needed. The reason for this sex difference in short working hours is as follows: compared to unhealthy women, unhealthy men are more likely to be involuntarily thrown into precarious jobs with short working hours [23]. In Korea, there are profound gender inequalities regarding wage and housework time. In 2016, men earned more than twice what women earned, and men’s housework time was far shorter than women’s (3.2 h vs. 19.5 h) [29]. Such concentrations on men’s wage and women’s housework are so rooted that unhealthy men and women tend to work at the workplace or household, respectively, if they can barely work due to poor health. A study on the association between precarious jobs and poor SRH only included men, which also supports our interpretation [30].

High educational level and related occupations (manager and professionals, e.g., executive and physician) were associated with poor SRH when working long hours, which can be attributed to the lack of leisure-time physical activity. This shortage is related to poor health [31] and SRH [32]. White-collar workers have relatively long leisure-time physical activity [33,34]; therefore, the reduction of leisure-time physical activity due to long working hours would be greater in high educational occupations. On the contrary, low educational level and related occupations (simple labor, e.g., cleaner, helper) were associated with poor SRH when working for short hours. This can be interpreted as follows: the unhealthy effect of precarious jobs demanding short working hours is found only among workers with low education. Low educational level is related to precarious jobs [35], which are associated with short working hours and poor SRH [23]. The present study verified this chain of relationships exposing the simple association between working hours and SRH.

Paid employees working short hours showed poor SRH as opposed to employers or self-employed workers; this can be attributed to the differences in autonomy in modulating working hours. Employers or self-employed workers desiring short work can modulate their working hours at will; however, the short working hours of some employees would be involuntarily decided by the employers. Despite the fact that non-fixed work schedules were widely researched [36,37], an association of SRH according to working hours in non-fixed workers did not appear in the present study. Thus, estimating the effects of working hours on SRH in non-fixed workers appears difficult in a cross-sectional study. A study reported no relationship between shift work and SRH [38]. Thus, a longitudinal study is required.

This study has several limitations. Firstly, owing to its cross-sectional design, the causality between working hours and SRH could not be fully explained; the probability of reverse causality cannot be negligible. Secondly, due to recall bias, which inevitably occurred due to a retrospectively conducted survey, the participants’ answers could be inaccurate to a certain extent. Finally, these findings cannot be extrapolated to all participants due to differences in the reliability of self-rated health according to SES, ethnicity, gender, and age [39]. Nevertheless, this study has several novel points. This study stratified various factors, including sex, SES, and working conditions, so that the trends of SRH by working hours according to workers’ social and working traits could be predicted delicately. Additionally, by restricting age and adjusting several health-related factors, we tried to minimize the healthy worker effect.

## 5. Conclusions

In conclusion, long and short working hours were associated with poor SRH. Men working short hours were at risk of poor SRH, while women working short hours were not, which is attributed to the gender inequalities of wage and housework. Highly educated workers’ long working hours and less-educated workers’ short working hours were associated with poor SRH, indicating that workers’ educational level is a distinct factor in predicting SRH according to working hours. Occupations associated with either high or low educational levels had the same involvement, thus supporting the predictability of educational level. Employers or self-employed workers did not have any risk of poor SRH with short working hours, whereas paid employees did. The SRH of non-fixed workers did not worsen with long or short working hours in contrast to fixed workers; this implies that both short and long working hours are associated with workers’ health, but the magnitudes differ according to sex, SES, and working conditions. Further research is needed to clarify the causal association between working hours and self-rated health. 

## Figures and Tables

**Table 1:** General characteristics of study participants by weekly working hours.

Characteristics	Total	Weekly Working Hours				*p*-Value
	(*N* = 25144)	<20 h	20–39 h	40–59 h	≥60 h	
	*N* (%)	*N* (%)				
**Total population**		2141 (8.5)	5616 (22.3)	13,515 (53.8)	3872 (15.4)	
**Sex**						
Men	13,157 (52.3)	578 (4.4)	2108 (16.0)	7940 (60.3)	2531 (19.2)	<0.001
Women	11,987 (47.7)	1563 (13.0)	3508 (29.3)	5575 (46.5)	1341 (11.2)	
**Age**						
20–29	3202 (12.7)	459 (14.3)	629 (19.6)	1829 (57.1)	285 (8.9)	<0.001
30–39	5673 (22.6)	312 (5.5)	1054 (18.6)	3524 (62.1)	783 (13.8)	
40–49	6856 (27.3)	437 (6.4)	1479 (21.6)	3922 (57.2)	1018 (14.8)	
50–59	6846 (27.2)	585 (8.5)	1710 (25.0)	3257 (47.6)	1294 (18.9)	
60–64	2567 (10.2)	348 (13.6)	744 (29.0)	983 (38.3)	492 (19.2)	
**Household income quartile**						
1st quartile	1762 (7.0)	318 (18.0)	558 (31.7)	623 (35.4)	263 (14.9)	<0.001
2nd quartile	5889 (23.4)	568 (9.6)	1382 (23.5)	2866 (48.7)	1073 (18.2)	
3rd quartile	8201 (32.6)	608 (7.4)	1723 (21.0)	4625 (56.4)	1245 (15.2)	
4th quartile	9292 (37.0)	647 (7.0)	1953 (21.0)	5401 (58.1)	1291 (13.9)	
**Education**						
Elementary school	2486 (9.9)	233 (9.4)	724 (29.1)	957 (38.5)	572 (23.0)	<0.001
Junior high school	2473 (9.8)	244 (9.9)	629 (25.4)	1045 (42.3)	555 (22.4)	
High school	9050 (46.0)	899 (9.9)	2040 (22.5)	4572 (50.5)	1539 (17.0)	
College	11,135 (44.3)	765 (6.9)	2223 (20.0)	6941 (62.3)	1206 (10.8)	
**Occupation**						
Simple labor	2880 (11.5)	426 (14.8)	884 (30.7)	1160 (40.3)	410 (14.2)	<0.001
Craft, plant and machine operator, and assembler	4385 (17.4)	121 (2.8)	692 (15.8)	2603 (59.4)	969 (22.1)	
Skilled agricultural, forestry, and fishery	1516 (6.0)	148 (9.8)	460 (30.3)	592 (39.1)	316 (20.8)	
Service and sales	5572 (22.2)	522 (9.4)	1407 (25.3)	2282 (41.0)	1361 (24.4)	
Clerk	4492 (17.9)	270 (6.0)	673 (15.0)	3295 (73.4)	254 (5.7)	
Manager and professional	6299 (25.1)	654 (10.4)	1500 (23.8)	3583 (56.9)	562 (8.9)	
**Type of work**						
Paid employee	17,272 (68.7)	1430 (8.3)	3585 (20.8)	10,447 (60.5)	1810 (10.5)	<0.001
Employer/self-employed	6451 (25.7)	612 (9.5)	1582 (24.5)	2562 (39.7)	1695 (26.3)	
Unpaid family worker	1421 (5.7)	99 (7.0)	449 (31.6)	506 (35.6)	367 (25.8)	
**Work schedule**						
Fixed	20,903 (83.1)	1,560 (7.5)	4446 (21.3)	11,877 (56.8)	3020 (14.4)	<0.001
Non-fixed	4241 (16.9)	581 (13.7)	1170 (27.6)	1638 (38.6)	852 (20.1)	
**Obesity**						
Underweight (<18.5)	944 (3.8)	122 (12.9)	224 (23.7)	494 (52.3)	104 (11.0)	<0.001
Normal (<25)	15,616 (62.1)	1419 (9.1)	3600 (23.1)	8356 (53.5)	2241 (14.4)	
Obese (≥25)	8584 (34.1)	600 (7.0)	1792 (20.9)	4665 (54.3)	1527 (17.8)	
**Smoking**						
Never	13,935 (55.4)	1598 (11.5)	3663 (26.3)	6957 (49.9)	1717 (12.3)	<0.001
Past	4719 (18.8)	252 (5.3)	843 (17.9)	2789 (59.1)	835 (17.7)	
Current	6490 (25.8)	291 (4.5)	1110 (17.1)	3769 (58.1)	1320 (20.3)	
**Drinking**						
None	4402 (17.5)	533 (12.1)	1228 (27.9)	1928 (43.8)	713 (16.2)	<0.001
Moderate	15,652 (62.2)	1323 (8.5)	3534 (22.6)	8640 (55.2)	2155 (13.8)	
Severe	5090 (20.2)	285 (5.6)	854 (16.8)	2947 (57.9)	1004 (19.7)	
**Hypertension**						
Not present	22,003 (87.5)	1843 (8.4)	4908 (22.3)	12,002 (54.5)	3250 (14.8)	<0.001
Present	3141 (12.5)	298 (9.5)	708 (22.5)	1,513 (48.2)	622 (19.8)	
**Diabetes**						
Not present	23,964 (95.3)	2024 (8.4)	5321 (22.2)	12,970 (54.1)	3649 (15.2)	<0.001
Present	1180 (4.7)	117 (9.9)	295 (25.0)	545 (46.2)	223 (18.9)	
**Depression**						
Not present	24,800 (98.6)	2086 (8.4)	5510 (22.2)	13,378 (53.9)	3826 (15.4)	<0.001
Present	344 (1.4)	55 (16.0)	106 (30.8)	137 (39.8)	46 (13.4)	
**Self-rated health**						
Good	21,767 (86.6)	1791 (8.2)	4836 (22.2)	11,893 (54.6)	3247 (14.9)	<0.001
Poor	3377 (13.4)	350 (10.4)	780 (23.1)	1622 (48.0)	625 (18.5)	

**Table 2:** Risk of poor self-rated health according to weekly working hours from multivariable logistic regression.

Characteristics	Weekly Working Hours			
	<20 h	20–39 h	40–59 h	≥60 h
	Odds Ratio (95% Confidence Interval)			
**Total population**	1.299 (1.140–1.481)	1.091 (0.992–1.200)	Reference	1.289 (1.163–1.430)
**Sex**				
Men	1.384 (1.080–1.772)	1.123 (0.963–1.309)		1.224 (1.064–1.407)
Women	1.098 (0.938–1.286)	0.955 (0.844–1.081)		1.401 (1.197–1.639)
**Household income quartile**				
1st quartile	1.318 (0.939–1.850)	1.276 (0.955–1.705)		1.301 (0.907–1.866)
2nd quartile	1.111 (0.861–1.435)	1.113 (0.924–1.339)		1.214 (0.997–1.478)
3rd quartile	1.274 (0.994–1.632)	0.971 (0.814–1.157)		1.134 (0.940–1.369)
4th quartile	1.221 (0.947–1.573)	1.000 (0.841–1.188)		1.462 (1.221–1.751)
**Education**				
Elementary school	1.579 (1.143–2.181)	1.156 (0.918–1.457)		1.072 (0.835–1.376)
Junior high school	1.433 (1.024–2.007)	1.001 (0.774–1.296)		0.990 (0.757–1.295)
High school	1.091 (0.872–1.366)	1.110 (0.943–1.306)		1.273 (1.072–1.512)
College	1.262 (0.992–1.605)	0.964 (0.815–1.141)		1.321 (1.092–1.598)
**Occupation**				
Simple labor	1.596 (1.199–2.124)	1.186 (0.932–1.509)		1.078 (0.792–1.469)
Craft, plant and machine operator, and assembler	1.222 (0.734–2.035)	1.049 (0.812–1.356)		1.187 (0.954–1.478)
Skilled agricultural, forestry, and fishery	1.073 (0.682–1.689)	1.101 (0.808–1.502)		1.216 (0.857–1.725)
Service and sales	1.180 (0.893–1.559)	1.007 (0.826–1.229)		1.219 (1.007–1.477)
Clerk	1.376 (0.936–2.022)	1.150 (0.878–1.506)		1.125 (0.754–1.680)
Manager and professional	1.248 (0.954–1.632)	0.977 (0.793–1.204)		1.371 (1.048–1.793)
**Type of work** *				
Paid employee	1.436 (1.226–1.682)	1.119 (0.995–1.259)		1.232 (1.064–1.427)
Employer/self-employed	1.047 (0.806–1.359)	0.991 (0.820–1.197)		1.251 (1.048–1.493)
**Work schedule**				
Fixed	1.405 (1.212–1.629)	1.080 (0.972–1.201)		1.290 (1.149–1.449)
Non-fixed	0.894 (0.661–1.208)	1.044 (0.834–1.308)		1.188 (0.939–1.502)

Adjusted for age, obesity, smoking, drinking, hypertension, diabetes, and depression. Variables for stratification are not adjusted. Bold implies statistically significant. * Unpaid family workers were included in all analyses, but their odds ratios (ORs) were excluded from this table due to their small sample size.
